# Evaluation of Different Drying Treatments with Respect to Essential Oil Components, Phenolic and Flavonoid Compounds, and Antioxidant Capacity of Ajowan (*Trachyspermum ammi* L.)

**DOI:** 10.3390/molecules29143264

**Published:** 2024-07-10

**Authors:** Zahra Sobatinasab, Mehdi Rahimmalek, Nematollah Etemadi, Antoni Szumny

**Affiliations:** 1 Department of Horticulture, College of Agriculture, Isfahan University of Technology, Isfahan 84156-83111, Iran; z.sobatinasab@ag.iut.ac.ir (Z.S.); etemadin@iut.ac.ir (N.E.); 2 Department of Food Chemistry and Biocatalysis, Wrocław University of Environmental and Life Sciences, 50-375 Wrocław, Poland

**Keywords:** ajowan, drying, essential oil, phenolic, flavonoid, antioxidant

## Abstract

Ajowan (*Trachyspermum ammi* L.) is considered a valuable spice and medicinal herb. In this study, the essential oil content and composition of the aerial parts of ajowan were investigated under different drying treatments (sun, shade, oven at 45 °C, oven at 65 °C, microwave, and freeze drying). Moreover, the phenolic content, flavonoid content, and antioxidant capacity of samples were also assessed. Fresh samples produced the highest essential oil content (1.05%), followed by those treated under sun (0.7%) and shade drying (0.95%). Based on gas chromatography–mass spectrometry (GC–MS), thirty compounds were determined in which thymol (34.84–83.1%), carvacrol (0.15–32.36%), *p*-cymene (0.09–13.66%), and γ-terpinene (3.12–22.58%) were the most abundant. Among the drying methods, freeze drying revealed the highest thymol content, followed by drying in a 45 °C oven. The highest TPC (total phenolic content) and TFC (total flavonoid content) were obtained in the fresh sample (38.23 mg TAE g^−^^1^ dry weight (DW)) and in the sample oven-dried at 45 °C (7.3 mg QE g^−^^1^ DW), respectively. Based on the HPLC results, caffeic acid (18.04–21.32 mg/100 gDW) and ferulic acid (13.102–19.436 mg/100 g DW) were the most abundant phenolic acids, while among flavonoids, rutin constituted the highest amount (10.26–19.88 mg/100 gDW). Overall, freeze drying was the most promising method of drying for preserving the phenolic (TPC) and flavonoid (TFC) compounds and oil components.

## 1. Introduction

In food science, different plant organs can be applied as spices, and during the drying process, the structure of plant tissues can be highly affected [1]. Drying is a crucial technique in food science for enhancing the shelf life and storage period of harvested plants. Nowadays, producing high-quality food products with consistent nutritional values and low microbe concentrations is of great importance in food science. Different drying processes can impact both metabolites and microbial accumulations in the final food products [2]. Drying can extend the shelf life of products by inhibiting microorganism growth and certain biochemical processes [2]. Among the metabolites, polyphenolic compounds, as well as essential oils, are considered the most relevant in most spices in terms of health benefits and taste properties. Essential oils and flavonoids are the most susceptible compounds in terms of processing, as drying can lead to changes in the structure of many aromatic components as well as a decrease in flavonoid compounds [3,4].

Medicinal plants are considered a valuable source of secondary metabolites [5]. Ajowan (*Trachyspermum ammi* L.) is an important annual spice of the Apiaceae family. It is a cross-pollinated medicinal plant with white flowers and small brownish fruit. The Eastern Mediterranean region is considered the main origin of ajowan [6]. This species is distributed in India and Central Asia [6]. Different pharmaceutical and food properties of ajowan have been reported, including anti-*candida* [6], antioxidant [7], and sedative properties [7] reported for ajowan seeds. Ajowan oil has also been applied in sanitary and pharmaceutical products such as toothpaste and cough syrup. It has also been used as a spice in food industries [8]. Some reports detail the effect of different drying methods on metabolite changes in various aromatic plants, including *Nepeta binaludensis* [9], *Mentha longifolia* [10], *Salvia officinalis* [11], *Daucus carota* [1], *cannabis sativa* [12], *Alpinia latila bris* [13], and *Origanum vulgare* [14]. Most of the previous reports focused mostly on the seed metabolites. However, the aerial parts (leaves and flowers) of ajowan have some medicinal and food properties due to the presence of compounds beneficial for health, like flavonoids [15].

Considering that there are no reports regarding the drying procedure in ajowan as a valuable spice, the aims of the present study were as follows: (1) to assess the yield and composition of essential oils during six drying treatments, viz. shade drying, oven drying at 45 and 65 °C, freeze drying, sun drying, and microwave drying to evaluate the alternations in polyphenolic compounds affected by different drying methods and (2) to elucidate the best drying treatment in terms of essential oil components based on multivariate analyses.

## 2. Results and Discussion

### 2.1. Moisture Content

**Figure 1:**
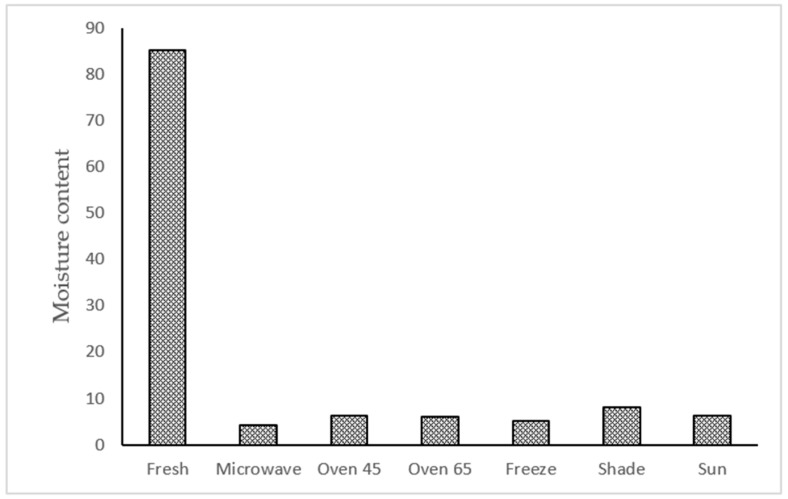
The moisture content of ajowan aerial parts affected by different drying treatments.

According to the results, the moisture content in the fresh sample was 85.19%, and the moisture decreased at a high rate in all drying treatments (Figure 1). Among the drying treatments, the lowest and the highest moisture ratio was obtained in microwave (4.18%) and shade drying (8.16%).

### 2.2. The Essential Oil Content Changes during Drying

**Figure 2:**
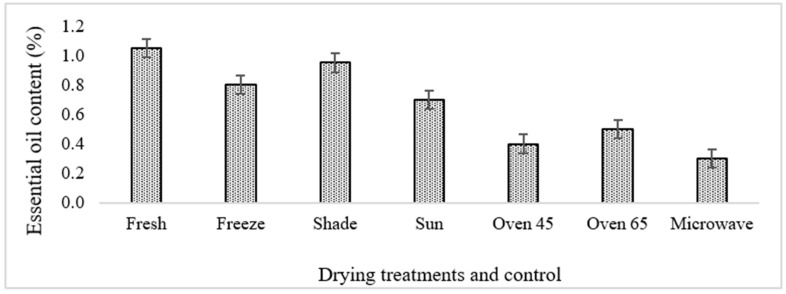
The variation in the essential oil content in ajowan using six different drying treatments.

The fresh sample of ajowan originating from aerial parts possessed a lower essential oil content in comparison with that reported in the seeds [16]. The essential oil content showed a high variation based on the drying methods (Figure 2). Most of the treatments led to an increase in the oil content compared to the fresh sample, while microwave drying showed a lower amount of oil compared to the fresh sample. The lowest and the highest oil content belonged to microwave-dried (0.3%) and fresh samples (1.05%). Regarding oil content, a similar trend was also revealed in *Thymus daenensis* [2,17], *Coriandrum sativum* [18], and *Stevia rebaudiana* [19], while some other studies reported different drying methods as the efficient ones to increase oil content [4,20,21]. Essential oil content changes during drying can be highly affected by the type of tissue, temperature, and time of drying [4]. In this study, freeze drying revealed the best results in terms of oil content. Microwave and shade drying showed the lowest amount of oil content. A loss of some volatile oil due to its diffusion during drying in the atmosphere, especially during shade drying, might be the main reason [2]. The nonuniformity in the heating process in microwave drying is considered the major drawback of this method and results in the formation of temperature gradients in the product, especially large-sized products [22].

Moreover, the type of compounds in the oil can affect the oil content during drying. For instance, some aromatic plants have more volatile compounds in comparison with others, and the volatiles are located in the superficial tissue and can easily diffuse into the atmosphere, while in some other plants, the oils are scattered in non-superficial secretory tissues that can highly affect the oil content. In the present investigation, the highest essential oil content was obtained in the fresh [1] and freeze-dried samples (0.8), followed by those undergoing shade drying (0.95) (Figure 2). Freeze drying has been considered an efficient drying procedure for preserving the aroma of plants because it uses low temperatures and has less aroma reduction in comparison with other methods [17].

### 2.3. The Effect of Drying Methods on Essential Oil Composition

Sixteen components were identified in the essential oil of ajowan using different drying methods (Table 1). The main components of the oil were thymol (from 34.84% after sun drying to 83.1% after oven drying at 65 °C), carvacrol (from 0.15% after shade drying to 32.36% after sun drying), *p*-cymene (from 0.09 after sun drying to 13.66% after oven drying at 45 °C), and γ-terpinene (from 3.12% in the fresh sample to 22.58% after freeze drying).

In the study of ajowan, the thymol content was elevated at a higher oven temperature, while the opposite trend was revealed in *Thymus vulgaris* [17]. This discrepancy in results may be due to the tissues of essential oil production. In the Lamiacaeae family, essential oils mostly accumulate in the secretory hairs located in the epidermis [23], while in the Apiaceae family, like ajowan, the oils mostly accumulate in secretory sacs, and consequently, oil release needs greater destruction of the tissues by using high temperatures in comparison with Lamiaceae plants like thyme.

Drying can greatly affect the tissue structure of plants. In fresh plants, essential oils are mostly stored on the surface of the leaves in the secretory trichomes located on leaves, roots, or bark [22]. Furthermore, during the drying process, the retention of oils in dried plants highly depends on the integrity of the oil glands in the dried plants [24]. Thus, trichome integrity during drying is considered a crucial factor for improving the oil of dried plants.

### 2.4. Correlation Analysis

According to Table 2, a positive correlation was observed between β-myrcene and α-terpinene (r = 0.93), γ-terpinene, and β-myrcene (r = 0.82), while some other compounds showed negative correlations, e.g., thymol and carvacrol (r = −0.68). Thymol revealed a negative correlation with γ-terpinene and *p*-cymene. γ-terpinene and *p*-cymene are considered precursors for thymol production [2].

### 2.5. Total Phenolic (TPC) and Flavonoid Content (TFC)

**Figure 3:**
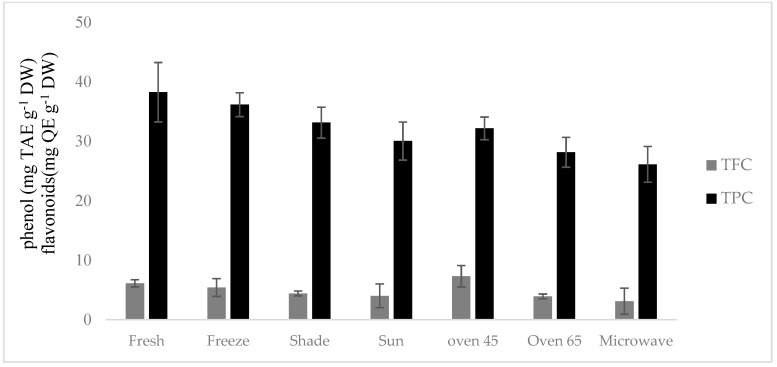
Total flavonoid content (TFC); total phenolic content (TPC).

The lowest and highest levels of TPC were obtained in the microwave-dried sample (26.11 mg TAE g^−1^ DW) and the fresh sample (38.23 mg TAE g^−1^ DW), respectively, while microwave drying (3.1 mg QE g^−1^ DW) revealed the lowest TFC and oven drying at 45 °C (7.3 mg QE g^−1^ DW) showed the highest TFC (Figure 3).

### 2.6. Alteration in Phenolic and Flavonoid Compounds during Drying

The polyphenolic pattern of the studied ajowan was highly affected by the drying treatments. Drying treatments increased the content of phenolic and flavonoid compounds (Table 3). High variation was observed based on different drying treatments. Among the compounds, caffeic acid (from 18.04 mg/100 g dry weight after oven drying at 65 °C to 21.32 mg/100 g dry weight after freeze drying), ferulic acid (13.10 mg/100 g dry weight after oven drying at 65 °C to 19.43 mg/100 g dry weight after freeze drying), and rutin (10.26 mg/100 g dry weight after oven drying at 65 °C to 19.88 mg/100 g dry weight after freeze drying) were the most abundant. Overall, freeze drying was the most promising method of drying for preserving the phenolic and flavonoid compounds. Similar results were reported in different plants, including *Coriandrum sativum* [18], *Fistulina antarctica* and *Ramaria patagonica* [25], *Daucus carota* [1], *Salvia officinalis* [26], *C. asiatica, A. vera* [27], *Alpinia latilabris* [13], and *lemon flavedo* [28].

The stability of phenolic compounds during drying is of great importance in food processing. Drying can lead to a loss of phenolic compounds, possibly due to oxidation [1]. The biological activity of flavonoids depends on the structure and substitution pattern of the hydroxyl groups. Among the flavonoids studied, rutin was less affected by the different drying methods. A similar trend was also observed in *Centella asiatica* [29]. Among the treatments, oven drying at 65 °C led to the highest decrease in polyphenolic compounds. This could be due to the degradation of phenolic compounds at high temperatures.

### 2.7. Antioxidant Activity

#### DPPH Method

**Figure 4:**
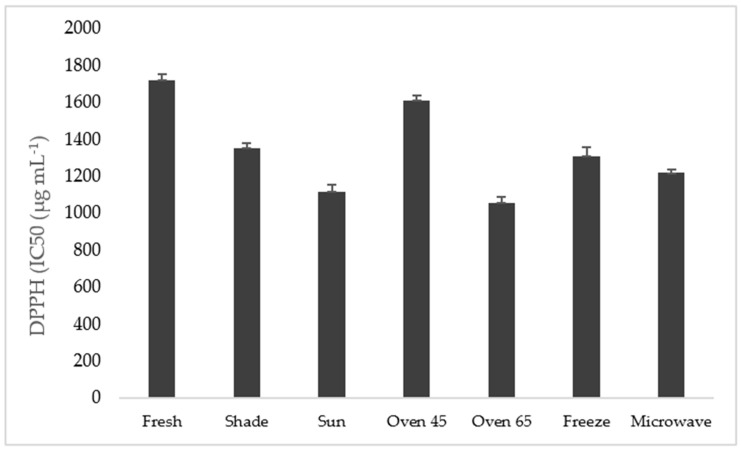
IC_50_ of different extracts based on DPPH assay comparing drying methods.

The antioxidant activity, measured by the DPPH assay and expressed in IC_50_, is shown in Figure 4. High antioxidant activity (low value of IC_50_) was obtained in the sample oven-dried at 45 °C and in the fresh sample, while low antioxidant activity was observed in the samples after oven drying at 65 °C and sun drying treatments.

The low antioxidant activity in the microwave 1100 µg mL^−1^ drying technique is indicated by the high value of IC_50_ in the sample oven-dried at 45 °C and the fresh sample (2123 µg mL^−1^ and 1986 µg mL^−1^), which significantly altered the antioxidant capacity of ajowan, either being raised or reduced (Figure 4).

### 2.8. Multivariate Analyses

**Figure 5:**
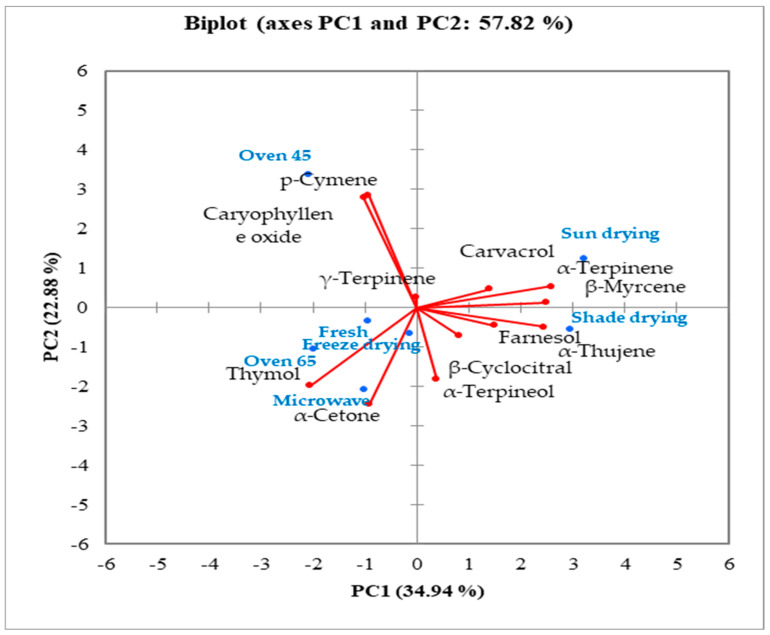
Biplot analysis of different drying treatments in the studied ajowan polyphenolic compounds.

Based on the biplot, essential oil components were categorized into three major groups (Figure 5). The main precursors of thymol biosynthesis, viz. *p*-cymene and γ-terpinene, were grouped in the oven drying at 45 °C group, while thymol, as the main compound, was classified in the oven drying at 65 °C group. Thus, based on the biplot, it might be hypothesized that temperatures lower than 50 °C can prepare the precursors for the increasing thymol content at higher temperatures.

**Figure 6:**
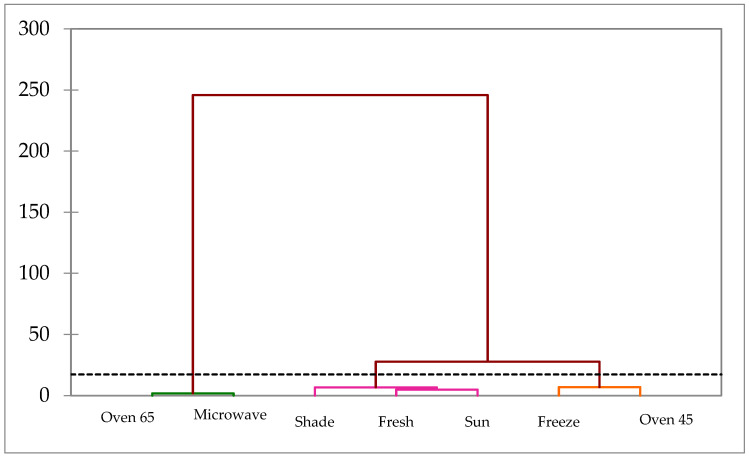
Cluster analysis of different drying treatments in ajowan polyphenolic compounds.

Regarding polyphenolic compounds, the cluster analysis illustrated three major groups (Figure 6). Group 1 included the sample undergoing microwave and oven drying at 65 °C that was separated from other treatments. Group 2 was composed of the fresh sample, and shade and sun drying treatments, while Group 3 comprised oven drying at 45 °C and freeze treatments.

**Figure 7:**
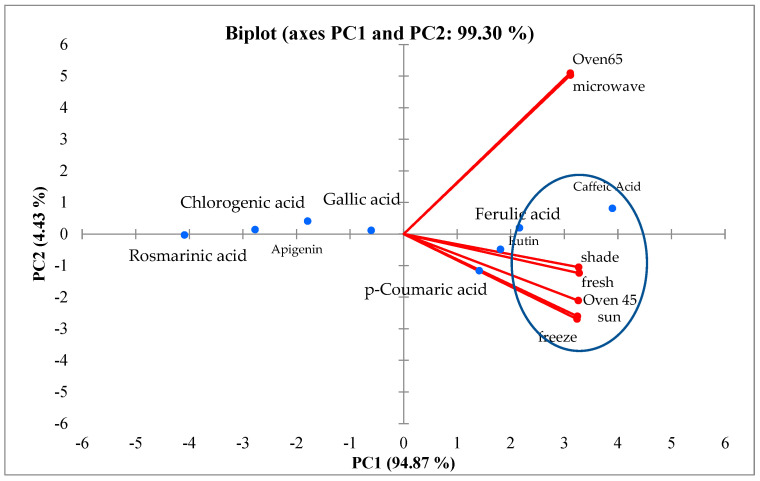
Biplot analysis of different drying treatments in ajowan polyphenolic compounds.

According to the results obtained from the PCA, the different drying methods are divided into two groups; the first includes microwave and oven drying at 65 °C. In general, these compounds (gallic acid, apigenin, rosmarinic acid, and chlorogenic acid) are less affected by different drying methods. The explained biplot analysis also confirmed the results in most cases. Based on the biplot analysis, oven drying at 65 °C and microwave drying led to an increase in caffeic acid and ferulic acid (Figure 7).

## 3. Materials and Methods

### 3.1. Plant Materials

Iranian ajowan (*Trachyspermum ammi* L.) seeds were obtained from the research institute of forests, range, and watershed management organization’s gene bank. Prof. Valiolah Mozaffarian used Flora Iranica [30] for plant identification, and the voucher specimen was submitted to the gene bank with 1085 as the herbarium number. The seeds were planted on 10 May 2023 on the research farm of the Isfahan University of Technology. The soil in which the seeds were planted was silty clay loam, pH = 7.38 and EC = 3.25 ds/m, Isfahan province, Iran (32°59′8 N and 50°24′43 E), at an altitude of 1900 m above sea level.

### 3.2. Drying Methods and Equipment

A fresh sample was used as a control treatment, and shade drying was carried out using natural airflow at 25 °C. For sun drying, the leaves were dried in direct sunlight at 30–40 °C for 48 h on 15 June 2023 in Isfahan, Iran. Oven drying was carried out in a ventilated oven at 45 and 65 °C, respectively, for 30 and 24 h. Microwave drying was carried out in a microwave oven (MA3884 VC, LG, Seoul, Republic of Korea) at 180 V and 800 W. The samples were placed on small plates. Freeze drying was performed for 24 h at −40 °C in a freeze dryer, Heto (Drywinner—Model DW8, Allerød, Denmark). The moisture content of the studied ajowan was evaluated in triplicate.

### 3.3. Essential Oil Extraction

Aerial parts (leaves and flowers) of ajowan were used to extract essential oils. For this purpose, 50 g of the samples was used for each hydrodistillation process. A Clevenger-style apparatus with a round-bottomed flask was used to isolate the EOs. A total of 400 mL of distilled water was used for a 5–6 h period, and then, the EOs were collected. Measurements were taken in triplicate, and the essential oil content was calculated based on the dry matter.

EO content (%) = volume of EO obtained (mL) × 100/mass of dry matter (g)

### 3.4. Essential Oil Analyses Using GC-MS

Oil components were analyzed using an Agilent 7890 gas chromatograph equipped with an HP-5MS 5% phenylmethylsiloxane capillary column (30 m × 0.25 mm, with a film thickness of 0.25 μm). Helium was used as the carrier gas at a flow rate of 2 milliliters per minute with a split ratio of 1:20. The initial oven temperature was set at 60 °C for 3 min, then increased at a rate of 3 °C per minute to 120 °C, and further raised to 300 °C at a rate of 15 °C min^−1^. The injector temperature was maintained at 300 °C. A mass detector, Agilent 5975 C, was used with the scanning conditions set at 39–400 *m*/*z*, 200 °C, and an electron ionization of 70 eV. The injection volume was 1 μL of a 1% solution of essential oils in cyclohexane (obtained from Sigma Aldrich company, Mashhad, Iran).

Compounds were determined using the HP-5MS column and retention indices (RIs) of *n*-alkanes (C_5_–C_24_) and compared with the available literature such as Willey (Chem Station data system, Santa Clara, CA, USA) and NIST 08 (National Institute of Standards and Technology, Gaithersburg, MD, USA) [31].

### 3.5. TPC

TPC was evaluated using the methodology described by Gharibi et al. [32]. Five grams of dried ajowan was extracted with 200 mL of 80% methanol and shaken for 10 h at 25 °C and 150 rpm, and this was repeated three times. The Folin–Ciocalteu method was applied for the evaluation of TPC [32]. The absorbance was assessed at 765 nm, and TPC was expressed as tannic acid equivalent per g of dry weight (DW) of the sample (TAEg^−1^DW).

### 3.6. TFC

TFC was measured using the aluminum chloride method [33]. Initially, 75 µL of the extract was mixed with 75 µL of a 5% NaNO_2_ solution. Subsequently, 150 µL of 10% AlCl_3_ was added, and the combination was allowed to incubate for 5 min before introducing 750 µL of NaOH (1 M). The absorbance was then read at 510 nm. Ultimately, TFC was quantified in quercetin equivalents per g of dry weight (QEg^−1^DW) of the extract.

### 3.7. Antioxidant Capacity

#### DPPH Assay

Tohidi et al. [23,34] developed the technique used to perform the DPPH assay. For this technique, 0.1 mL of plant extracts of the aerial part was mixed with 5 mL of 0.1 mM methanol DPPH solution at concentrations of 50, 100, 300, and 500 μg mL^−1^. Absorbances were read at 517 nm. Lastly, the EC_50_ value for antioxidant capacity was determined. The equations are illustrated in Supplementary Material S1, Figures S1–S12.

### 3.8. Polyphenolic Compound Determination Using HPLC

HPLC analysis was performed on extracts made of finely powdered aerial parts of ajowan following the protocol outlined by Gharibi et al. [35], using an Agilent 1090 HPLC model. To prepare the samples, 50 mL of methanol (80%) (HPLC grade, Merck, St. Louis, MO, USA) was combined with 2.5 g of ajowan plant material (extract of aerial parts after drying) and then agitated overnight at 150 rpm. The extracts were filtered using a 0.22 μm nylon Acrodisc filter. Calibration curves were established for polyphenolic standards, such as rutin, apigenin, *p*-coumaric acid, caffeic acid, rosmarinic acid, and chlorogenic acid, all with a purity exceeding 98% (Phyto lab, Vestenbergsgreuth, Germany) (Supplementary Material S2).

HPLC analysis was performed using an analytical column (250 mm × 4.6 mm, 5 μm, Waters Symmetry C18 column with a pre-column of 10 mm × 4 mm, Waters Corp., Milford, MA, USA). A total of 20 μL of the extract was injected for analysis. The mobile phase gradient consisted of solvents A (0.1% aqueous formic acid) and B (0.1% formic acid in acetonitrile). The gradient program involved a linear increase from 10% to 26% of solvent B (*v*/*v*) over 40 min, reaching 65% of solvent B at 70 min and ultimately 100% of solvent B at 75 min, all while maintaining a flow rate of 0.8 mL min^−1^. Chromatograms were examined between 200 and 400 nm. Phenolic compounds were identified by comparing retention time (RT) and UV spectra with reference standards. Peak areas were determined using calibration curves for each standard used in the quantification process. Finally, the data were reported in milligrams per 100 g of dry weight in the samples.

### 3.9. Statistical Analyses

Each test was run in three replicates. Data collected were reported as means with standard deviation (SD). The graph plotting software utilized was Statgraphics Software (ver. 18), and SAS JMP version 11 was used for clustering analysis (HCA).

## 4. Conclusions

The present study provides new insights into drying effects on the secondary metabolites of ajowan aerial parts for the first time. With respect to essential oil content, freeze and sun drying showed the best and the worst efficiency, respectively. Moreover, freeze drying led to the preservation of the major component of the oil with the best efficiency. Furthermore, in the presented research, phenolic and flavonoid alternation was evaluated during drying, where fresh samples and freeze drying provided the most promising results, while microwave and oven drying at 65 °C showed the worst efficiency. Overall, based on multivariate analyses, freeze drying and oven drying at 45 °C revealed the best results for essential oil content and composition, and freeze drying and fresh samples had the best results in terms of polyphenolic compounds. Finally, based on the analyzed compounds, aerial parts of ajowan can be introduced as potential additives in further food industries with an optimal drying procedure.

## Figures and Tables

**Table 1:** Essential oil compounds of ajowan by different drying treatments.

Compounds	RI	Fresh	Microwave	Oven at 45	Oven at 65	Freeze Drying	Shade Drying	Sun Drying
α-Thujene	931	0.33 ± 0.1	0.12 ± 0.4	0	0	0.2 ± 0.2	0.26 ± 0.3	0.26 ± 0.1
β-Myrcene	993	0.12 ± 0.2	tr	tr	tr	0.2 ± 0.03	0.26 ± 0.4	0.26 ± 0.2
α-Terpinene	1016	0.18 ± 0.3	tr	tr	tr	tr	0.12 ± 0.3	0.14 ± 0.1
***p*-Cymene**	1026	3.43 ± 0.1	tr	13.66 ± 0.4	tr	tr	0.83 ± 0.2	0.09 ± 0.1
**γ-Terpinene**	1057	3.12 ± 0.2	3.66 ± 0.2	9.32 ± 0.2	7.32 ± 0.3	22.58 ± 0.1	6.940 ± 0.2	7.07 ± 0.3
***cis*-Verbenol**	1140	1.85 ± 0.04			tr			
α-Terpineol	1193	0.2 ± 0.2	0.10 ± 0.2	0	tr	0	0.09 ± 0.1	0
β-cyclocitral	1213	2.9 ± 0.1	0.07 ± 0.3	0	0.13 ± 0.1	0.46 ± 0.3	2.9 ± 0.3	0
**Thymol**	1292	73.9 ± 0.4	74.61 ± 0.2	58.13 ± 0.2	83.1 ± 0.1	71.49 ± 0.2	56.56 ± 0.3	34.84 ± 0.3
Carvacrol	1299	3.28 ± 0.1	12.66 ± 0.08	2.12 ± 0.3	2.11 ± 0.3	1.09 ± 0.03	0.15 ± 0.1	32.36 ± 0.2
Isopulegol	1461				0.37 ± 0.1			
Caryophyllene oxide	1584	tr	0	2.82 ± 0.3	0	0.13 ± 0.2	0.22 ± 0.3	0.15 ± 0.1
Geranyl isovalerate	1611		0.1 ± 0.2			0.07 ± 0.2		
Farnesol	1684	6.29 ± 0.4	0.08 ± 0.1	1.02 ± 0.1	0.37 ± 0.07	1.27 ± 0.3	32.36 ± 0.05	0
Heptacosane	1703					0.2 ± 0.1		
Eicosane	1980	tr				0.14 ± 0.1		

tr: trace; RI: Retention index.

**Table 2:** Correlation between phenolic compounds.

	α-Thujene	β-Myrcene	*p*-Cymene	Thymol	Carvacrol	Phytol	γ-Terpinene	α-Terpinene
α-Thujene	1							
β-Myrcene	0.936998	1						
*p*-Cymene	−0.40461	−0.31638	1					
Thymol	−0.61857	−0.67438	−0.19879	1				
Carvacrol	0.439234	0.345773	−0.22365	−0.6875	1			
Phytol	−0.5383	−0.43233	−0.22226	0.368282	−0.21601	1		
γ-Terpinene	0.292278	0.389469	0.043388	0.041667	−0.25062	−0.39092	1	
α-Terpinene	0.782905	0.823407	−0.21967	−0.83298	0.57266	−0.31823	−0.16242	1

**Table 3:** Phenolic and flavonoid compounds of ajowan aerial parts affected by drying.

	RT	Fresh	Freeze	Sun	Oven 45	Oven 65	Shade	Microwave
Gallic acid	5.13	10.00 ± 0.4	13.10 ± 0.2	9.11 ± 0.06	12.12 ± 0.3	7.96 ± 0.4	11.02 ± 0.5	8.11 ± 0.2
Chlorogrnic acid	13.416	6.03 ± 0.3	7.12 ± 0.5	5.89 ± 0.04	6.15 ± 0.6	4.11 ± 0.2	5.13 ± 0.4	4.12 ± 0.4
Caffeic acid	14.12	21.00 ± 0.5	21.32 ± 0.1	19.15 ± 0.3	20.51 ± 0.04	18.04 ± 0.4	20.10 ± 0.5	19.06 ± 0.06
*p*-coumaric acid	27.11	16.12 ± 0.5	19.14 ± 0.03	17.45 ± 0.1	18.100 ± 0.1	8.020 ± 0.3	16.23 ± 0.3	7.81 ± 0.2
Rutin	28.412	18.12 ± 0.4	19.88 ± 0.06	16.19 ± 0.05	17.12 ± 0.1	10.26 ± 0.06	15.89 ± 0.3	11.12 ± 0.1
Ferulic acid	29.14	19.43 ± 0.2	16.86 ± 0.04	16.61 ± 0.4	18.19 ± 0.5	13.10 ± 0.3	15.14 ± 0.1	14.12 ± 0.4
Rosmarinic acid	35.905	4.11 ± 0.3	3.00 ± 0.5	2.84 ± 0.04	3.42 ± 0.5	1.02 ± 0.4	3.123 ± 0.3	1.15 ± 0.05
Apigenin	57.110	8.63 ± 0.33	7.340 ± 0.06	7.10 ± 0.4	7.79 ± 0.3	7.05 ± 0.5	8.89 ± 0.06	6.21 ± 0.03

RT: Retention Time.

## Data Availability

Data are contained within the article and Supplementary Materials.

## Supplementary Materials

The following supporting information can be downloaded at https://www.mdpi.com/article/10.3390/molecules29143264/s1, Figure S1. The equation used for calculation of p-coumaric acid content. Figure S2. The equation used for calculation of chlorogenic acid content caffeic acid. Figure S3. The equation used for calculation of caffeic acid content Gallic acid. Figure S4. The equation used for calculation of Gallic acid content. Figure S5. The equation used for calculation of rutin content. Figure S6. The equation used for calculation of Apigenin content. Figure S7. The equation used for calculation of ferulic acid content. Figure S8. The equation used for calculation of Rosmarinic acid content. Figure S9. Standard peak of Gallic acid and ferulic acid. Figure S10. Standard peak of Chlorogrnic acid and Rutin and p- coumaric acid. Figure S11. Standard peak of Rosmarinic acid. Figure S12. Standard peak of caffeic acid and some other phenolic and flavonoid standards.
