# Supplementary material for: Evaluation of Different Drying Treatments with Respect to Essential Oil Components, Phenolic and Flavonoid Compounds, and Antioxidant Capacity of Ajowan (Trachyspermum ammi L.)
Source: Molecules. 2024 Jul 10;29(14):3264. doi: 10.3390/molecules29143264 (PMC11279657; doi:10.3390/molecules29143264)
Supplement: Supplementary file 1 [file molecules-29-03264-s001.zip › molecules-3069023-supplementary.pdf]

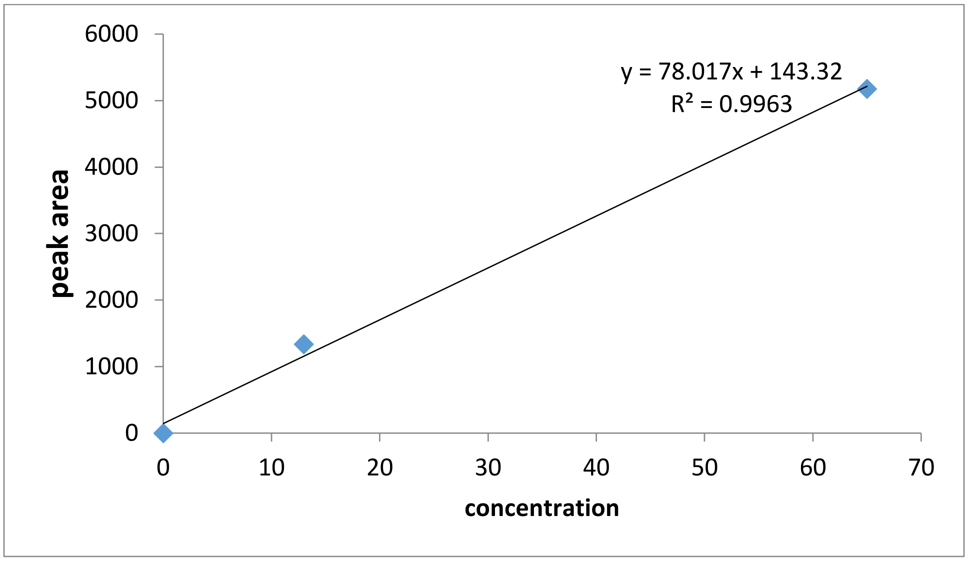

Figure S1. The equation used for calculation of *p*-coumaric acid content

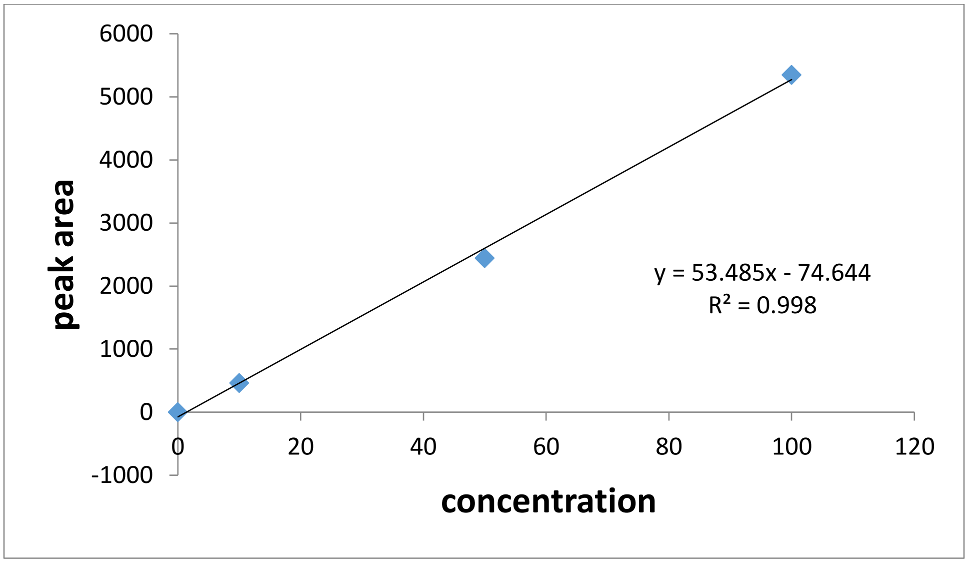

Figure S2. The equation used for calculation of chlorogenic acid content caffeic acid

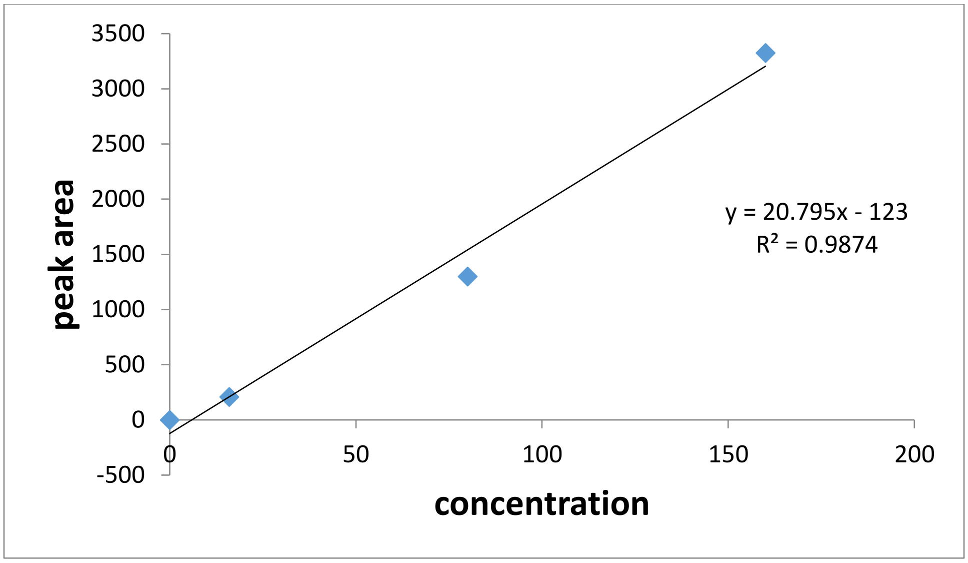

Figure S3. The equation used for calculation of caffeic acid content Gallic acid.

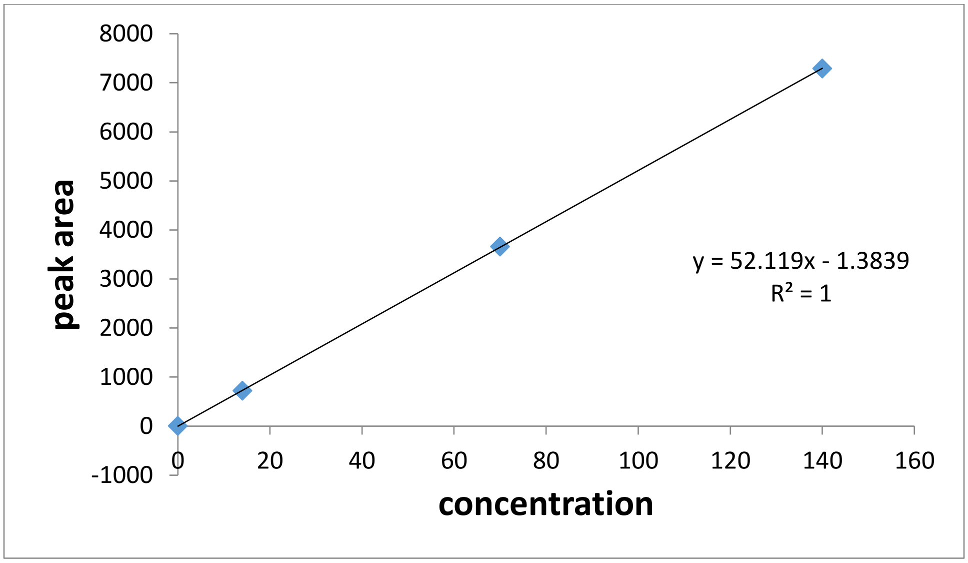

Figure S4. The equation used for calculation of Gallic acid content

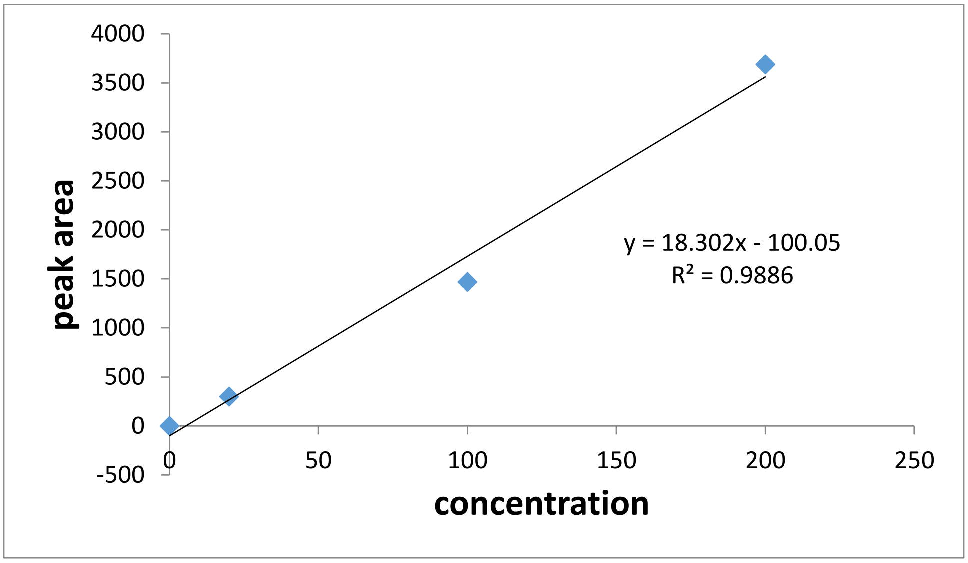

Figure S5. The equation used for calculation of rutin content

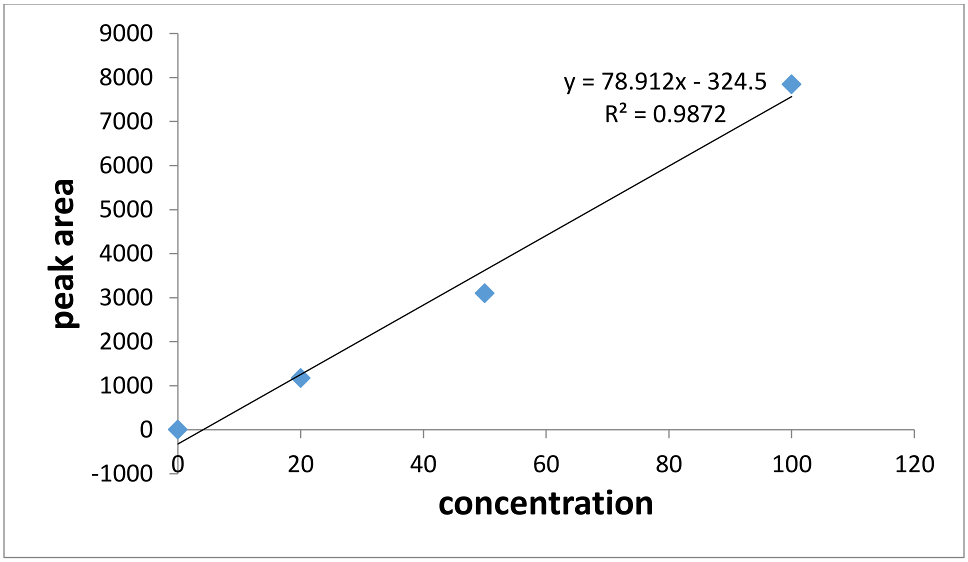

Figure S6. The equation used for calculation of Apigenin content

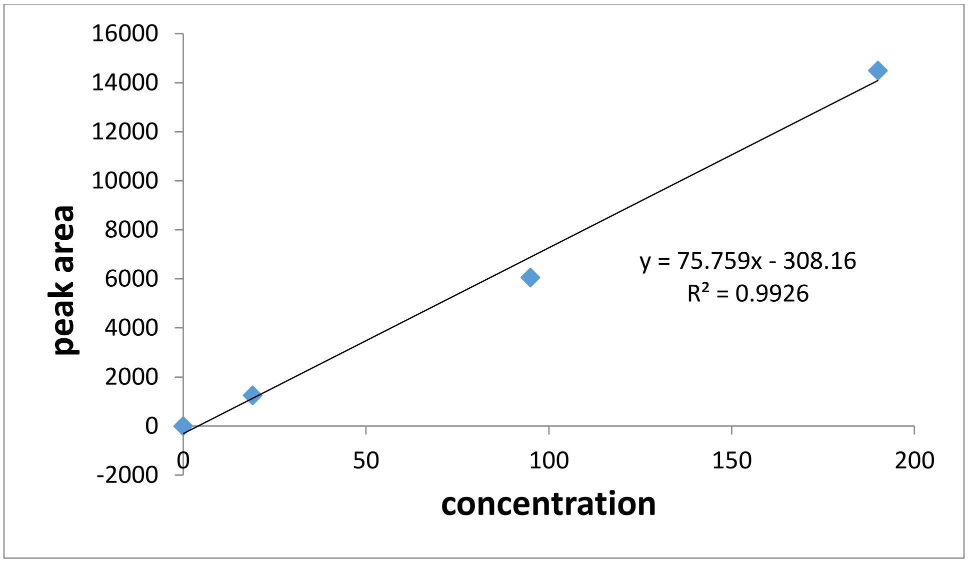

Figure S7. The equation used for calculation of ferulic acid content.

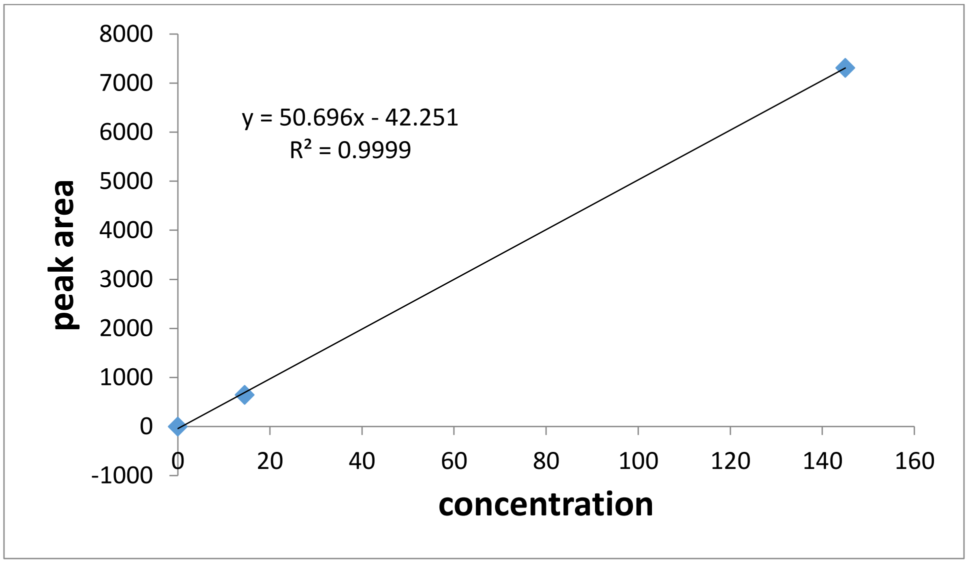

Figure S8. The equation used for calculation of Rosmarinic acid content.

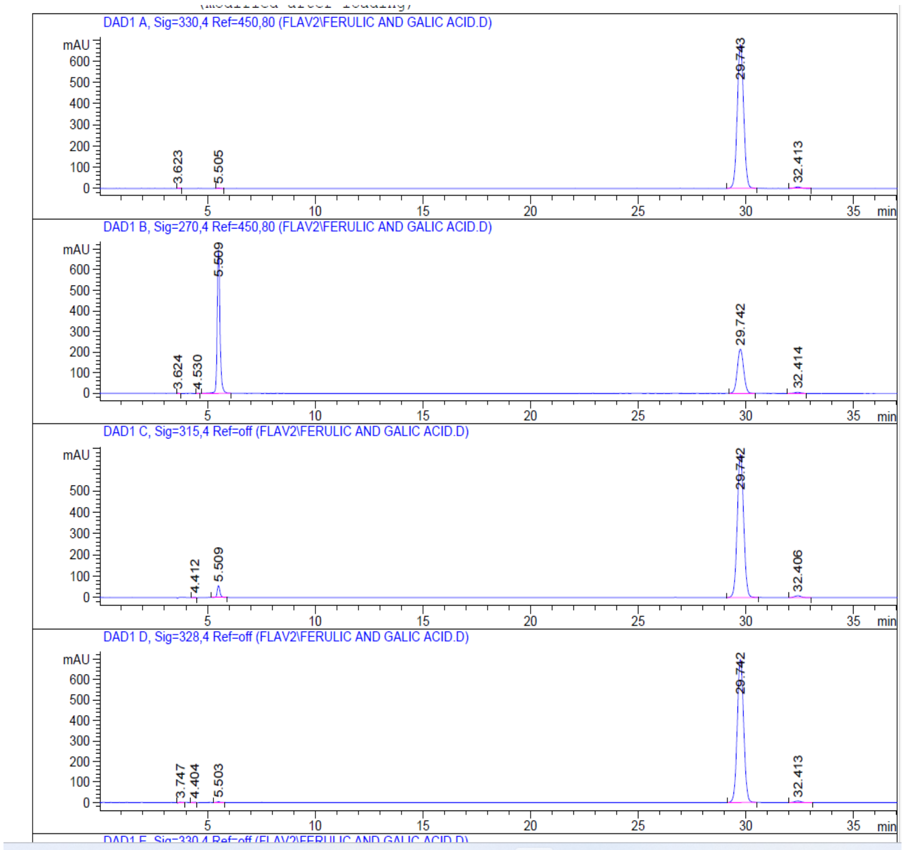

Figure s9. Standard peak of Gallic acid and ferulic acid

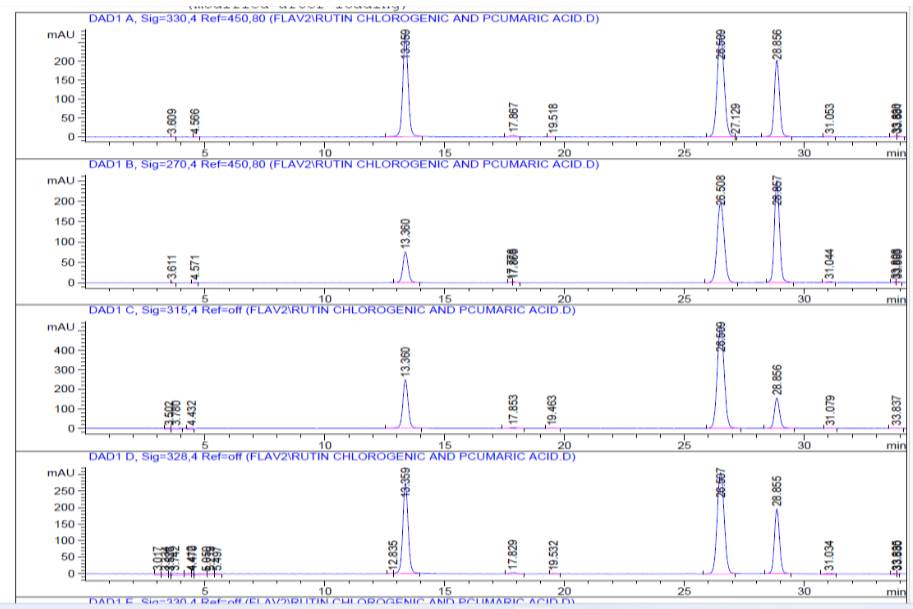

Figure s10. Standard peak of Chlorogrnic acid and Rutin and *p*- coumaric acid

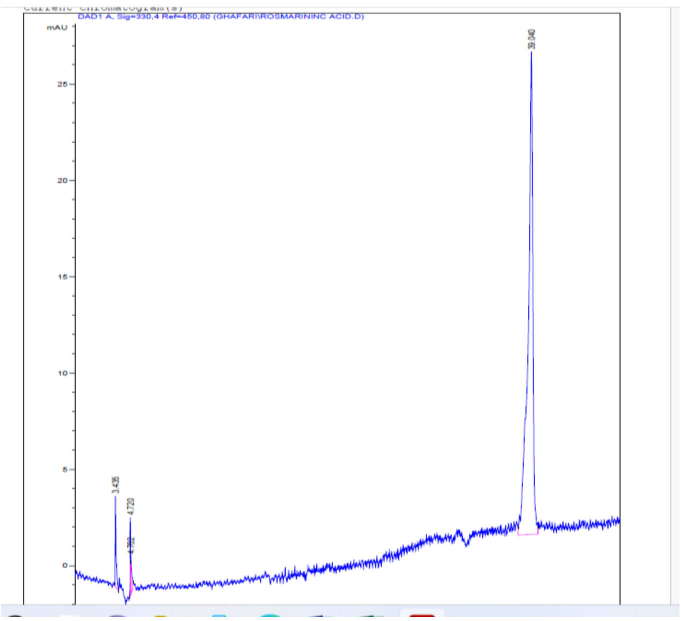

Figure s11. Standard peak of Rosmarinic acid

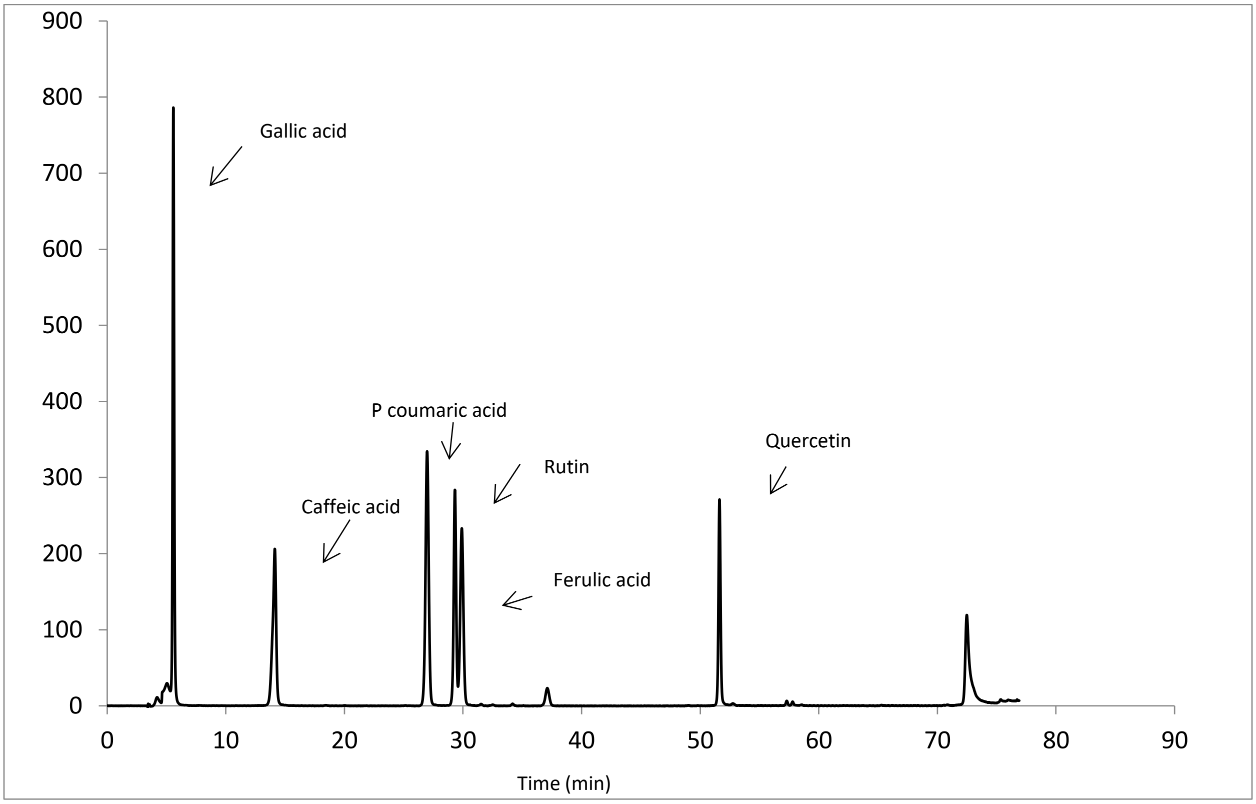

Figure S12.Standard peak of caffeic acid and some other phenolic and flavonoid standards.

Supplementary material 2. Antioxidant activity calculations:

For calculating the antioxidant activity, when readings in 517nm were done, the inhibition percentage of the samples was calculated according to the following formula:

$\%inhibition= A_B - A_A / A_B \times 100$

where, A_A and A_B are the absorbance values of the DPPH radical in the presence of the plant extract sample and the control, respectively. The inhibition percentage was plotted versus the sample concentration and 50% of the inhibitory concentration (IC_{50}) of the DPPH values was defined by linear regression analysis.
